# Expression of hub genes of endothelial cells in glioblastoma-A prognostic model for GBM patients integrating single-cell RNA sequencing and bulk RNA sequencing

**DOI:** 10.1186/s12885-022-10305-z

**Published:** 2022-12-06

**Authors:** Songyun Zhao, Wei Ji, Yifan Shen, Yuansheng Fan, Hui Huang, Jin Huang, Guichuan Lai, Kemiao Yuan, Chao Cheng

**Affiliations:** 1 Department of Neurosurgery, Wuxi People’s Hospital Affiliated to Nanjing Medical University, No. 299 Qing Yang Road, 214023 Wuxi, Jiangsu China; 2 Department of Epidemiology and Health Statistics, School of Public Health, Chongqing Medical University, Yixue Road, 400016 Chongqing, China; 3 Department of Oncology, Traditional Chinese Medicine Hospital of Wuxi, No.8, West Zhongnan Road, 214071 Wuxi, China

**Keywords:** GBM, scRNA-seq, Risk score, Prognostic model, Immune cell infiltration

## Abstract

**Background:**

This study aimed to use single-cell RNA-seq (scRNA-seq) to discover marker genes in endothelial cells (ECs) and construct a prognostic model for glioblastoma multiforme (GBM) patients in combination with traditional high-throughput RNA sequencing (bulk RNA-seq).

**Methods:**

Bulk RNA-seq data was downloaded from The Cancer Genome Atlas (TCGA) and The China Glioma Genome Atlas (CGGA) databases. 10x scRNA-seq data for GBM were obtained from the Gene Expression Omnibus (GEO) database. The uniform manifold approximation and projection (UMAP) were used for downscaling and cluster identification. Key modules and differentially expressed genes (DEGs) were identified by weighted gene correlation network analysis (WGCNA). A non-negative matrix decomposition (NMF) algorithm was used to identify the different subtypes based on DEGs, and multivariate cox regression analysis to model the prognosis. Finally, differences in mutational landscape, immune cell abundance, immune checkpoint inhibitors (ICIs)-associated genes, immunotherapy effects, and enriched pathways were investigated between different risk groups.

**Results:**

The analysis of scRNA-seq data from eight samples revealed 13 clusters and four cell types. After applying Fisher’s exact test, ECs were identified as the most important cell type. The NMF algorithm identified two clusters with different prognostic and immunological features based on DEGs. We finally built a prognostic model based on the expression levels of four key genes. Higher risk scores were significantly associated with poorer survival outcomes, low mutation rates in IDH genes, and upregulation of immune checkpoints such as PD-L1 and CD276.

**Conclusion:**

We built and validated a 4-gene signature for GBM using 10 scRNA-seq and bulk RNA-seq data in this work.

**Supplementary Information:**

The online version contains supplementary material available at 10.1186/s12885-022-10305-z.

## Introduction

Due to its confined and locally aggressive growth, GBM is one of the most prevalent malignant tumors globally, with a significant morbidity and fatality rate [1]. It is also the most common primary intracranial tumor [2]. The prognosis of GBMs is dismal, with less than 5% of affected patients surviving > 5 years at the time of diagnosis. With the research advancements, remarkable results have been achieved in exploring the molecular pathogenesis of glioma, such as isocitrate dehydrogenase (IDH) status [3] and O6-methylguanine DNA methyltransferase promoter (MGMTp) methylation [4]. Diagnoses, categorization systems, and precise therapy have all improved due to these findings. However, although IDH mutations help individuals with gliomas live longer, gliomas with IDH mutations are prone to frequent return [5]. Therefore, further research is essential for identifying new molecular targets, prognostic assessment work, and developing therapeutic options. Only four medications, including bevacizumab, temozolomide, lomustine, and carmustine, have been authorized by the US Food and Drug Administration (FDA) to treat GBM [6]. Although these adjuvant drugs and surgical treatments have improved the prognosis of glioma patients to some extent, the overall survival (OS) of patients is still very low [7], which is partly because the mechanisms of the tumor microenvironment and immune evasion are not fully understood and high-grade gliomas are spatially and temporally heterogeneous. In addition, different cells have different mutational characteristics [4]. Most important is the blood-brain barrier (BBB), a dynamic interface between blood and brain tissue that selectively prevents the passage of substances. The effectiveness of antitumor chemotherapeutic agents is hampered by the blood-brain barrier, which strictly regulates the homeostasis of the central nervous system [8].

In recent years, many studies have used traditional bulk RNA sequencing data to explore potential prognostic markers for GBM and improve our understanding of tumorigenesis and progression. For example, a prognostic model was developed based on 5 ferroptosis-related genes to predict survival and response to immunotherapy in GBM patients [9]. In addition, a prognostic model of GBM was also constructed based on three angiogenesis-related bases [10]. However, these prognostic characteristics are based on traditional RNA-seq, and because GBM is a highly heterogeneous tumor, these approaches cannot detect exact cellular and molecular alterations considering that bulk RNAseq mostly represents the “average” expression of all cells in the sample [11].

Endothelial cells (ECs) regulate vascular functions, such as permeability, endocytosis, and angiogenesis [12]. Abnormal vascular proliferation and vascular system abnormalities are the most characteristic features of GBM [13]. Vascular abnormalities promote tumor cell invasion by inducing hypoxia, thereby exacerbating GBM progression [14]. In addition, GBM vascular leakage can lead to edema [15]. In the 1970s, Professor Folkman proposed that tumor growth and metastasis are dependent on angiogenesis [16]. Therefore, inhibition of angiogenesis can be a therapeutic strategy for tumor treatment. Meanwhile, ECs are key cellular components of the BBB, and abnormal vascular development in gliomas is associated with their unique gene expression [17]. In recent years, targeting pro-angiogenic genes for tumor treatment and prevention of tumor expansion has gained increasing interest among researchers [18].

Single-cell RNA-seq (scRNA-seq) is nowadays used as a new technology for sequencing genes in different cell types that can provide a more in-depth understanding of cell-specific information. Given the advantages of single-cell sequencing, markers of GBM endothelial cells can be identified by integrating scRNA-seq and conventional RNA-seq and then can be used to construct prognostic models of GBM patients with external validation cohorts to verify their risk stratification ability. Herein, we explored potential neoangiogenic pathways in GBM after controlling for phenotypic differences between ECs of GBM and normal brain tissue ECs at the single-cell level. Finally, through the constructed 4-gene signature, we observed the prognostic and immunological characteristics of different risk subgroups of the population. Our findings provide deeper insights into the characteristics of ECs in GBM and potential prognostic biomarkers to design rational treatment regimens and optimized drugs.

## Materials and methods

### Raw data acquisition

We downloaded 10X of scRNA-seq data from the GSE162631 dataset, with 10,446, 11,821, 15,352, 16,750, 21,415, 15,008, 13,653 and 15,122 cells per sample. Due to the large number, we extracted one-tenth of these cells for subsequent studies, such as pathways and cellular communication. A large number of RNAseq data, mutation data, and clinicopathological features of TCGA-GBM were downloaded from the UCSC Xena website. In addition, we downloaded normal brain tissue expression data for GTEx from the UCSC Xena website (https://xena.ucsc.edu/). The final externally validated gene expression profiles and clinical data of patients with GBM were obtained from the China Glioma Genome Atlas (CGGA) data portal (http://www.cgga.org.cn/). Detailed clinical characteristics of patients in the TCGA and CGGA databases are summarized in Table S1.

### scRNA-Seq data processing and analysis

The 10× scRNA-seq data were processed as follows: (1) R package “Seurat” was used to convert the 10× scRNA-seq data into Seurat objects [19, 20]; (2) original counts were checked for quality by calculating the proportion of mitochondrial or ribosomal genes and eliminating cells with low quality; (3) after quality control, “FindVariableFeatures” function was used to screen the top 2000 highly variable genes; (4) based on 2000 genes for principal component analysis (PCA), uniform manifold approximation and projection (UMAP) [21] was used for dimensionality reduction and cluster identification; (5) using the “Find All Markers” function, log2 [Foldchange (FC)] was set to 0.3 and min, and pct was set to 0.25 to identify markers in different clusters; (6) the “SingleR” package was used for clustering annotations to identify different cell types [22]. In addition, the R package “ReactomeGSA” [23] was used to perform functional enrichment analysis of the identified hub cell types. We used the “analyze_sc_clusters” function to perform the enrichment analysis and extracted the results by “pathways”. The “monocle” package [24] scans cell trajectories and pseudotime distributions and reduces dimensionality using the “DDRTree” approach. Next, we calculated the contribution of genes to cell growth by using the BEAM statistical approach, and the top 100 genes were chosen for display. Cell-cell communication analysis and network visualization were finally performed using the “CellChat” [25] and “patchwork” software packages.

### Identification of key co-expression modules using WGCNA

Weighted gene co-expression network analysis (WGCNA, weighted correlation network analysis) is a systems biology method for identifying gene relationship patterns across samples. In the present study, we used the ‘WGCNA’ package in R to construct an expression data map of TCGA-GBM differential genes, which we then used to identify gene sets that vary synergistically in the GBM cohort and to identify biomarker genes based on gene set endogeneity and the association between gene sets and phenotypes. The appropriate soft threshold (power) for the TCGAGBM cohort was determined using the function “pickSoftThreshold”. Next, aij = |Sij|β (aij: adjacency matrix between gene i and gene j, Sij: similarity matrix, which is obtained by Pearson correlation of all gene pairs, β: soft power value) was used to calculate the matrix composed of weighted correlation values between genes and genes, i.e., the adjacency matrix. Finally, a hierarchical clustering dendrogram of dissimilarity (1-TOM) matrix was produced to compute the correlation between modules, where modules with strong correlation coefficients were selected as candidates for correlation with clinical features, and further analysis was performed. Studies with a more detailed description of the WGCNA method have been reported [26].

### Sample clustering based on non-negative matrix decomposition algorithm

Non-negative matrix decomposition (NMF) can be used to classify GBM patients into different subtypes: first, the sample is clustered using the R package “NMF” package, after which patients are classified into different subtypes based on parameters such as copulas and dispersions. Finally, a consensus heat map is generated based on the above optimal number of clusters to observe the distribution characteristics among different subtypes. Then, we also explored the relationship between different subgroups and OS. In addition, the MCP counter algorithm was used to estimate the infiltration of immune cells between different subgroups. Finally, we investigated the association between subpopulations and the six immune subtypes reported in previous studies [27].

### Identification of DEGs and functional enrichment analysis

Differential expression analysis of DEGs was performed for the TCGA cohort and different subgroups using the R software “limma” package with | log2FC |>1.0 and FDR < 0.05 as thresholds. In addition, we performed GSEA using Gene Set Enrichment Analysis (GSEA) software 4.1.0 (http://www.gsea-msigdb.org/gsea/index.jsp) to identify significantly enriched pathways between the low- and high-risk groups. *P* < 0.05 and FDR < 0.25 were considered thresholds for statistical significance. Results were visualized by the “gridExtra”, “grid” and “ggplot2” R software packages. In addition to this, functional enrichment analysis was performed by the “clusterProfiler” package in the R software, including the Kyoto Encyclopedia of Genes and Genomes (KEGG) and Gene Ontology (GO) analysis.

### Prognostic model construction and validation

The 169 GBM samples with survival information from the TCGA dataset were used as the training set for constructing the prognostic risk model, and the 388 GBM samples with survival information from the CGGA dataset were used for external validation. The r package “sva” was also used to eliminate batch effects between the TCGA and CGGA data and build an accurate model. Next, we matched the differential gene mRNA expression profiles from the TCGA-GTEx cohort to the WGCNA results.

Lasso Cox regression analysis was performed using the “glmnet” R package to minimize over-fitting prognostic characteristics and narrow down the genes that could predict OS. Multivariate Cox regression analysis was used to analyze the genes discovered using the Lasso method. The expression of each gene and the accompanying regression coefficients were used to create risk scores for each patient, and risk models for important genes were built in the TCGA cohort by weighting the estimated cox regression coefficients [28]. The risk score formula was Ʃ (ð × Expi), where ð was the corresponding regression coefficient and Expi represented the expression value of each gene. Based on the risk score formula, patients were divided into low-risk and high-risk groups using the median risk score as the cut-off point. The optimal cut-off point for survival analysis was determined using the ‘survcutpoint’ function in the R package ‘survminer’. A log-rank test was used to evaluate the difference in survival rates between the two groups, and Kaplan-Meier (K-M) survival curves were plotted.

In addition, the nomogram model was created using the R package “rms”.The receiver operating characteristic (ROC) curve was completed using the R package “survival ROC” and the corresponding area under the ROC curve (AUC) was measured to assess the sensitivity and specificity of the relevant characteristics.

### Mutation landscape, immune cells infiltration, and immune checkpoint between high- and low-risk groups

Two waterfall plots were generated using the ‘oncoplot’ function in the R package ‘maftools’ to explore the detailed mutations between the high-risk and low-risk groups. Each glioma sample’s immune and stromal fraction were assessed using the R package ‘ESTIMATE’, which indicates how many immune and stromal components are present in vivo. The tumor-infiltrating immune cells dataset was downloaded from TIMER 2.0 (http://timer.cistrome.org). TIMER, CIBERSORT, quantTIseq, MCP-counter, xCELL, and EPIC algorithms were also compared. In addition, we investigated the correlation between risk scores and immune checkpoint genes and tumor mutational load (TMB), which were visualized using the R software ‘ggplot2’ package.

### Prediction of immunotherapeutic response and evaluation of drug sensitivity

The Immune Cell Abundance Identifier (ImmuCellAI) is a computational method used to predict immune checkpoint responses based on the abundance of immune cells, specifically different T-cell subpopulations, which was released in 2020 [29]. TCIA (Cancer Immunome Atlas) is an online program that gives full immunogenomic analysis findings. The immunophenotype Score is a quantitative measure of tumor immunogenicity that ranges from 0 to 10 (IPS). Immune checkpoint inhibitor (ICI) response can be predicted using IPS [30]. The “prophytic” R package was used to compute the half-maximal inhibitory concentration (IC50) of samples from the high and low-risk score groups to analyze the risk score for predicting responsiveness to chemotherapy and molecular medicines.

### Statistical analysis

All analyses were performed using R version 4.1.1, 64-bit6, and its support package. Kaplan-Meier survival analysis and the log-rank test were used to calculate prognostic values and compare patient survival in different subgroups in each dataset. The non-parametric Wilcoxon rank sum test was used to test the relationship between the two groups for continuous variables. Kruskal-Wallis test was used for comparisons between more than two groups. LASSO regression and Cox regression analyses were used for predictive model development. Clinical characteristics of the high and low-risk groups were screened for prognostic variables using univariate and multivariate Cox regression (R package ‘survival’). Correlation coefficients were examined using spearman correlation analysis. A *P* value < 0.05 was considered statistically significant.

## Results

### scRNA-seq and cell typing of normal and glioblastoma brain samples

**Fig. 1:**
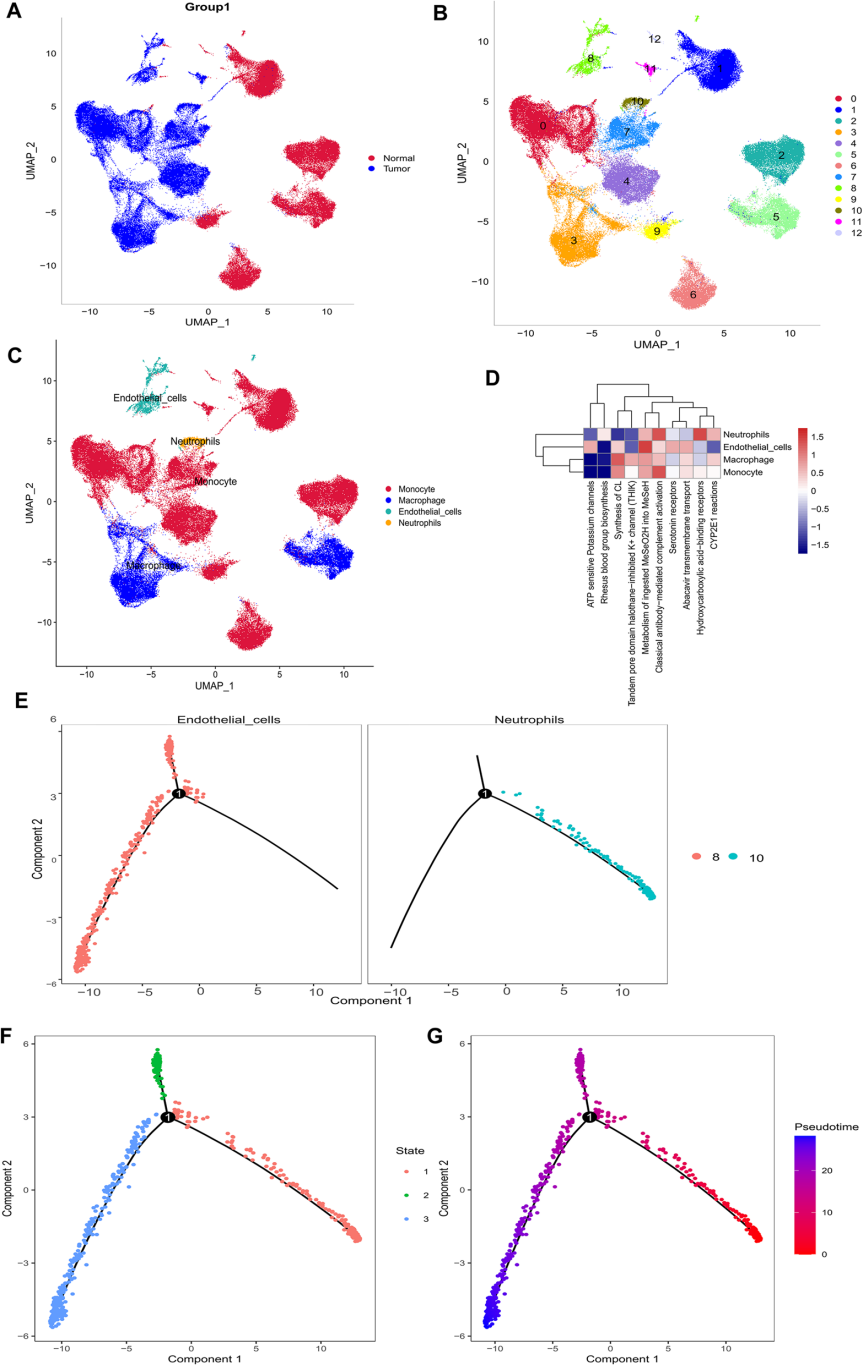
Different cluster annotations and cell type identification in GBM10 × scRNA-seq data. **A-C **Cluster annotation and cell type identification by UMAP. **D** Functional enrichment analysis of identified hub cell types using the “ReactomeGSA” package. **E-G** Cell trajectory and pseudo-time analysis for the identified hub cell types. GBM, glioblastoma multiforme; scRNA-seq, single cell RNA sequencing; UMAP, uniform manifold approximation and projection

We downloaded 10X scRNA-seq data from the GSE162631 dataset for four GBM and four normal samples (single cell suspensions of CD 31 + cells enriched for magnetically activated cell sorting (MACS)), where 102,412 cells were identified after cell quality control (QC) (Fig. S1A). FindVariableFeatures function was used to screen for highly variable genes based on expression data after normalization. The first 2000 highly variable genes are shown in Fig. S1B. After PCA and UMAP downscaling analysis, we identified 13 different cell clusters (Fig. 1A, B), after which we used the “SingleR” package for cluster annotation and UMAP to visualize the downscaled cell types. In addition, we identified four cell types, including ECs, monocytes, macrophages, and neutrophils (Fig. 1C). After applying Fisher’s exact test, ECs were identified as the most important cell type, and ReactomeGSA functional enrichment analysis showed that these cell types were mainly involved in classical antibody-mediated complement activation, transmembrane transport, and serotonin receptor and cardiolipin synthesis (CL) (Fig. 1D).

Next, the “monocle” R package was used to determine the cell trajectories and pseudotime distributions of two cell types that significantly differed in tumor and normal samples. We observed neutrophils corresponding to state 1 and ECs corresponding to states 2 and 3 (Fig. 1E-G).

Finally, we calculated the contribution of genes during cell development and selected the top 100 genes for visualization (Fig. S2A). Cell-cell communication networks were inferred by calculating the likelihood of communication (Fig. S2B). In addition, we predicted the cell-cell communication network for the relevant ligand receptors, finding that MSTN-TGFBR1-ACVR2A (Fig. S2C), WNT7B-FZD4-LRP6 (Fig. S2D), and others had a crucial role in the communication network of ECs .

### Identification of DEGs in bulk RNA-Seq data

**Fig. 2:**
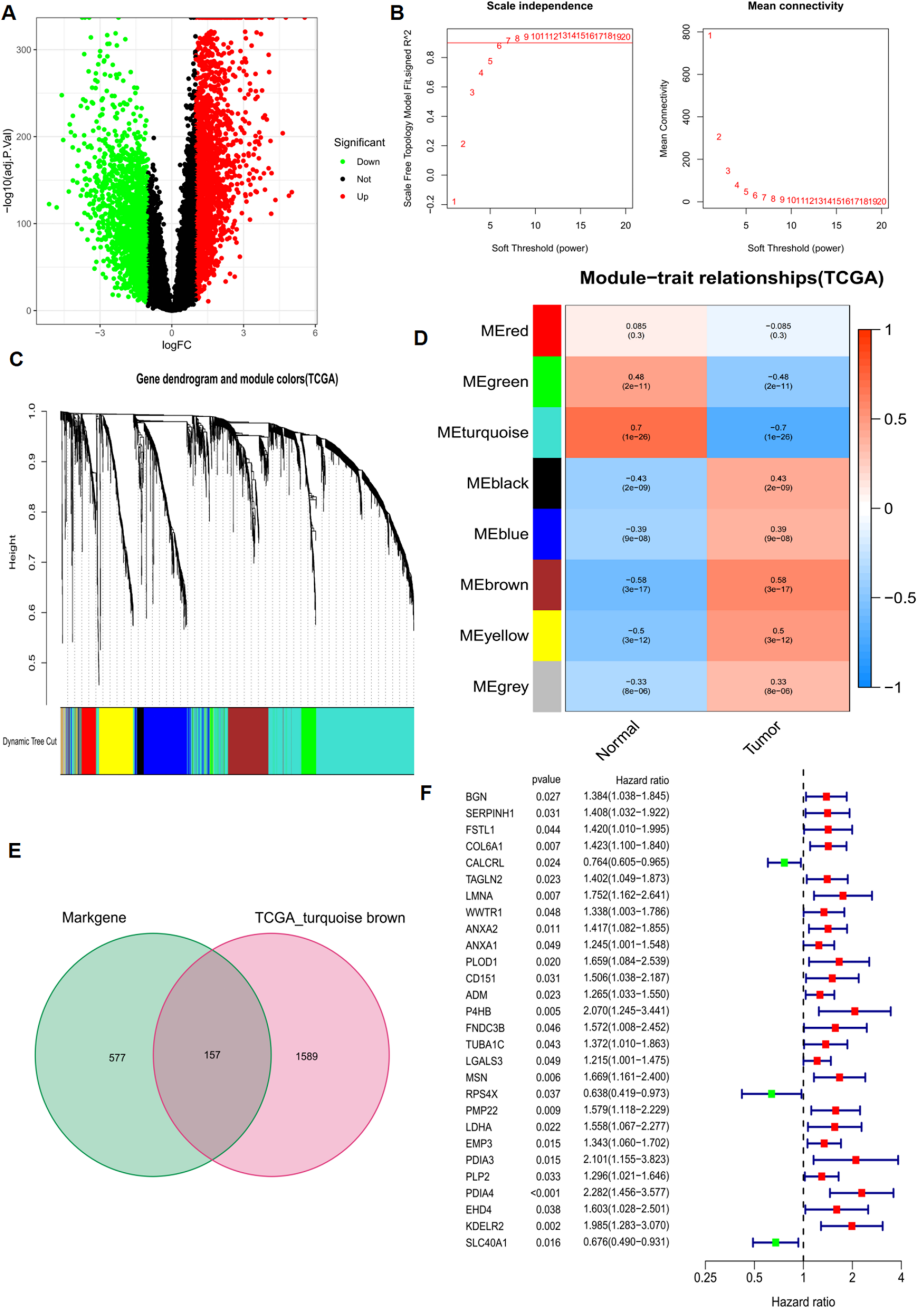
TCGA cohort differential analysis and WGCNA identification of hub genes in GBM development. **A **Volcano plot of up-and down-regulated DEGs in the TCGA-GTEx cohort. **B **Scale-free fit indices for soft threshold powers. Soft threshold powerβin WGCNA was determined by the scale-free R2 (*R2* = 0.90). The left panel shows the relationship between β and R2. The right panel shows the relationship between soft threshold power β and average connectivity. **C **Deg tree diagram based on clustering of different metrics. **D **Heat map illustrating the correlation between different gene modules and clinical features (normal vs. tumor). **E **Venn diagram between WGCNA module genes and endothelial cell marker genes. **F **Forest plot of the results of one-way cox analysis of 157 intersecting genes. DEGs, differentially expressed genes; TCGA, cancer genome atlas; GBM, glioblastoma multiforme; WGCNA, weighted gene correlation network analysis

We performed a differential analysis of tumor and normal tissues from the TCGA-GBM and GTEx cohorts, identifying 3911 DEGs. Of these, 2021 genes were up-regulated in tumors and 1901 genes were down-regulated (Fig. 2A).

Next, we used WGCNA to identify DEGs involved in the development of GBM associated with the TCGA cohort. In the process of co-expression network construction, we observed that the soft thresholding powerβwas 6 when the fit index of scale-free topology reached 0.90 (Fig. 2B, C). Eight modules were identified based on the average linkage hierarchical clustering and the soft thresholding power (Fig. 2C, D). Based on correlation coefficients and p-values, we observed that turquoise and brown modules were significantly associated with GBM development. We eventually took the intersection of marker genes of ECs and module genes of WGCNA and selected 157 genes to construct an expression matrix for further analysis (Fig. 2E).

Finally, we performed a univariate Cox regression analysis to identify potential prognostic factors for GBM in the TCGA cohort. A total of 28 genes were identified as being prognostically associated (Fig. 2F).

### Different molecular subtypes identification

**Fig. 3:**
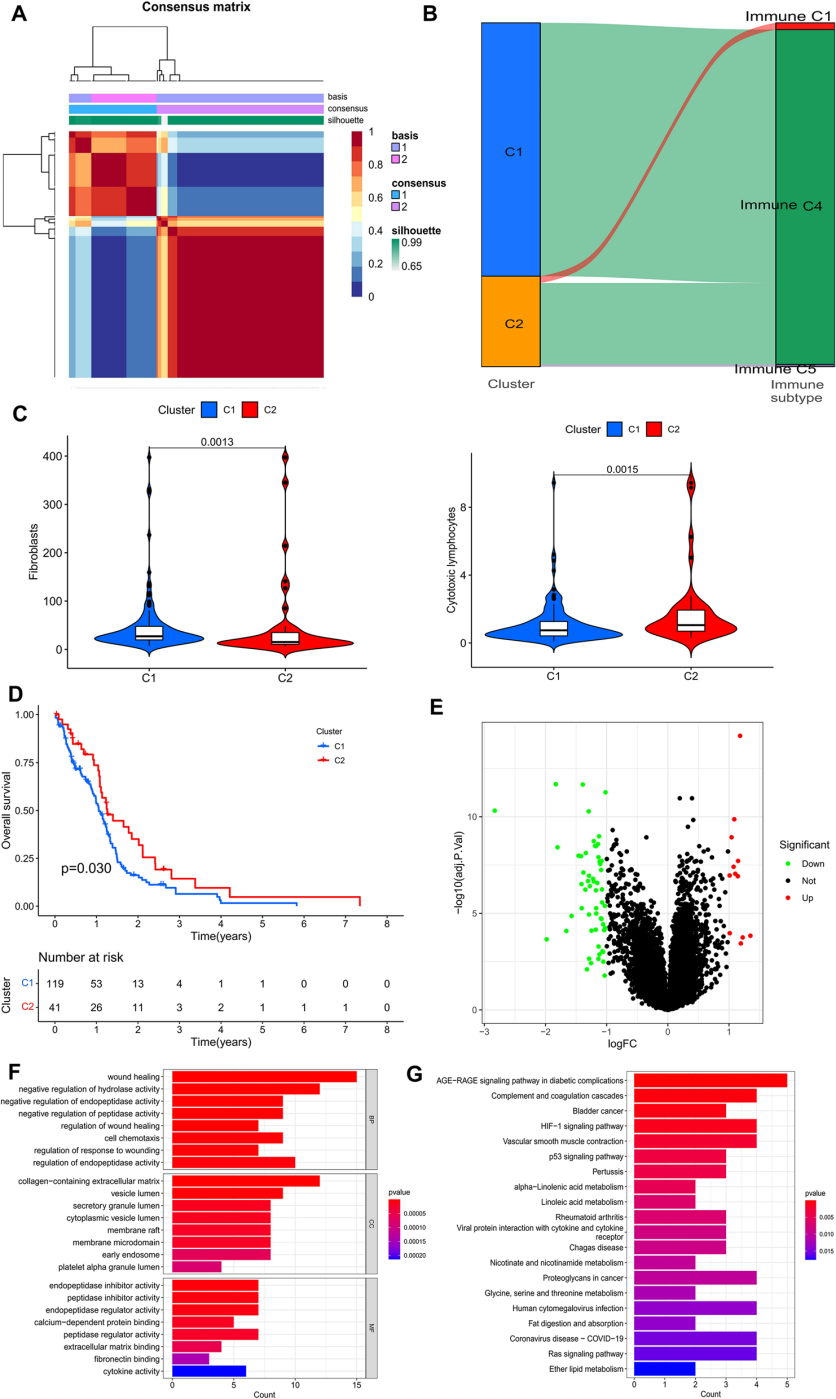
Identification of different subtypes. **A **The NMF algorithm identified two different subtypes. **B **Sankey plots show the association between different subtypes and immune subtypes. **C **Differences in TME between different subtypes. **D **Kaplan-Meier curves for overall survival in different GBM subgroups (log-rank test, *P* value = 0.03). **E **Heat map of the top 50 genes with the largest | log2FC | for different subtype differences analysis. **F, G **GO and KEGG enrichment analysis of DEGs. NMF: non-negative matrix decomposition; OS: overall survival; KEGG: Kyoto Encyclopedia of Genes and Genomes; GO: Gene Ontology

Based on the results of the univariate analysis, all patients were divided into two groups using the NMF algorithm (Fig. 3A). Sankey plots were used to investigate the relationship between different immune subtypes and groupings. The tumor samples were divided into different immune subtypes according to the GSVA enrichment score of 5 immune gene sets, i.e., wound healing, macrophages, lymphocyte, IFN-gamma, TGF-beta, and each immune subtype representing a specific immune microenvironment. The results showed that all patients in group 1 were classified as immune C4 (lymphocyte depleted) subtype. Due to the malignant nature of gliomas, most of the patients in group 2 were also classified as immune C4 (lymphocyte depleted) subtype, while only a few were classified as C1 (wound healing) subtype and immune C6 (TGF-beta dominant) subtype (Fig. 3B). The results showed that patients in group 2 had better OS compared to patients in group 1 (Fig. 3D). The MCPcounter algorithm was used to estimate the infiltration of immune cells in different clusters. The level of infiltration of cytotoxic lymphocytes was significantly higher in cluster 2; however, fibroblasts’ infiltration level was higher in group 1 (Fig. 3C).

After differential analysis of gene expression between the two groups, 56 genes were down-regulated in cluster 2 and 12 genes were up-regulated in cluster 2 (Fig. 3E). Finally, the R package “clusterProfiler” was used to perform GO and KEGG enrichment analysis in these DEGs, which were associated with a variety of items, including “wound healing” and “negative regulation of hydrolase activity” in the biological processes (BP) category, “collagen-containing extracellular matrix” in the cellular component (CC) category, and “endopeptidase inhibitor activity” in the molecular function (MF) category (Fig. 3F). They were also associated with HIF-1, P53, and signaling pathways in diabetes (Fig. 3G). HIF-1 confers survival to glioma cells, and it drives angiogenesis [31]. P53 is an oncogene whose mutation can affect the secondary GBM [32].

### Prognostic model construction and validation

**Fig. 4:**
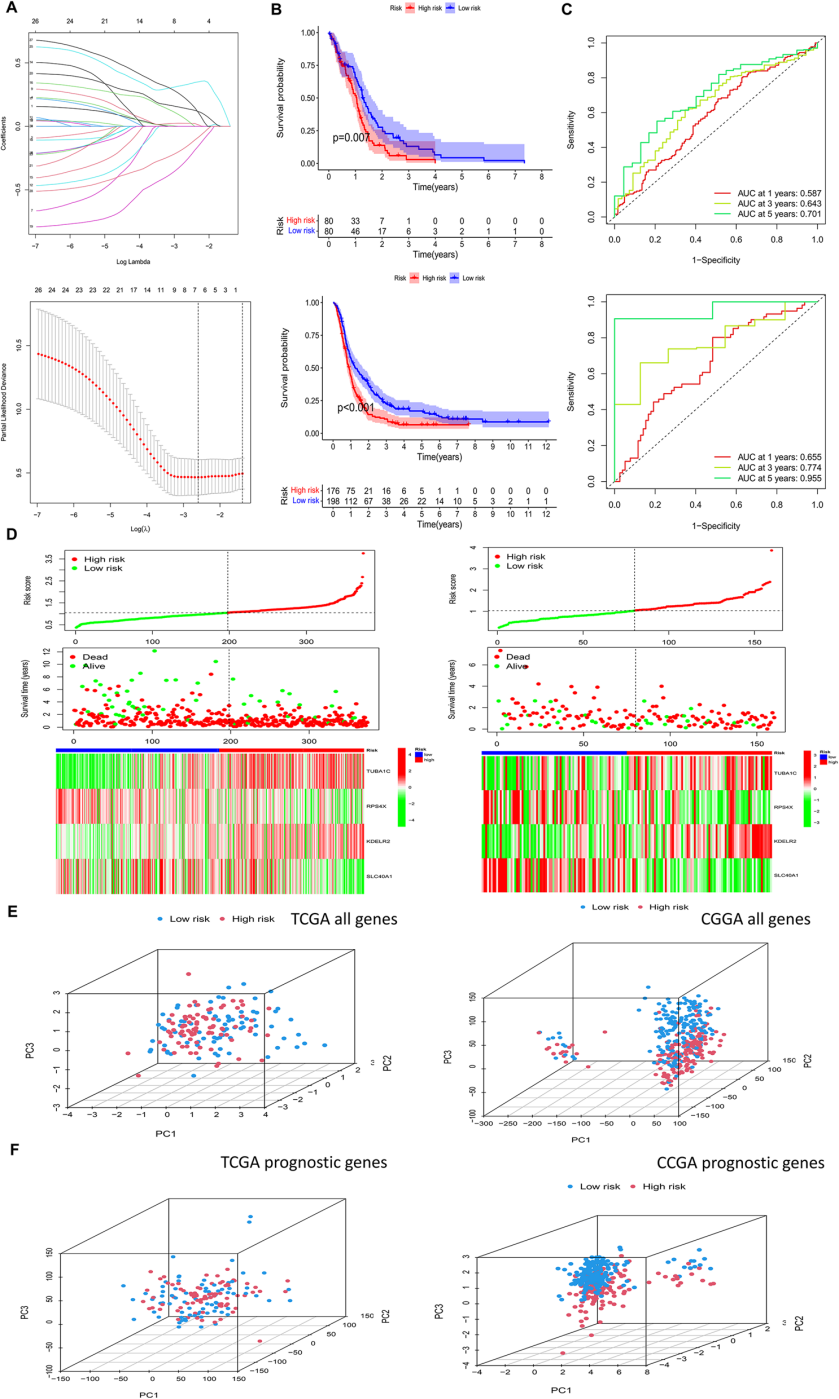
Development and validation of a prognostic model for GBM patients. **A **LASSO analysis with 10-fold cross-validation identified four prognostic genes. **B**, **C **Survival curves and ROC curves for evaluating the risk stratification ability and predicting the constructed risk models for the TCGA and CGGA cohorts. **D **Risk maps were used to illustrate the survival status of each sample in the TCGA and CGGA cohorts; heat maps represent the differences in expression of each gene in the risk groups. **E**, **F **Principal component analysis between the high- and low-risk groups in TCGA and CGGA entire set. GBM, glioblastoma multiforme; DEGs, differentially expressed genes; LASSO, minimal absolute shrinkage and selection manipulation; ROC, subject operating characteristic curve

We performed LASSO regression analysis on 28 prognostic genes (Fig. 2F), which can effectively reduce features in high-dimensional data and optimize predictors of clinical outcomes, identifying seven genes (ANXA2, TUBA1C, RPS4X, PMP22, PDIA4, KDELR2, and SLC40A1) at this step (Fig. 4A). Ultimately, by multivariate Cox analysis, four genes were identified as independent prognostic factors, including TUBA1C, RPS4X, KDELR2, and SLC40A1. Based on their coefficients, we calculated risk scores using the following formula: risk score = (expression level of TUBA1C*0.41)+(expression level of RPS4X*- 0.65)+(expression level of KDELR2*0.60)+(expression level of SLC40A1*-0.39). All patients were divided into high and low-risk groups according to the median value of the risk score. Survival curves showed that patients in the high-risk group had worse OS compared to those in the low-risk group (Fig. 4B, *P* < 0.001). Furthermore, the risk score performed well in predicting OS for these individuals in the TCGA cohort (Fig. 4C; AUCs for 1, 3, and 5-year OS: 0.655, 0.774, and 0.955). Similar results were observed in the CGGA cohort (Fig. 4B, C, AUC for 1-, 3-, and 5-year OS: 0.587, 0.643, and 0.701). The risk graph shows detailed survival outcomes for each patient in the TCGA cohort and the CGGA external validation cohort, with patients in the high-risk group mostly having poor prognostic outcomes. The heat map shows the difference in expression of the four genes in the models in the risk group (Fig. 4D), with TUBA1C and KDELR2 having higher expression in the tumor tissues, while RPS4X and RPS4X had the opposite tendency. In summary, the endothelial hub gene risk model had the best prognostic efficacy in GBM patients.

Next, we performed principal component analysis (PCA) to further validate the grouping ability of the four DEGs. PCA was performed to demonstrate the differences between the high and low-risk groups based on the prognostic characteristics of the whole gene expression profile and the expression profile classification of the four model genes. The results showed that the expression of the entire gene was diffusely distributed in both risk groups (Fig. 4E), while the expression of the four DEGs included in this prognostic risk model was well divided into two different risk clusters (Fig. 4F).

### Clinical features between the high-risk group and low-risk group

**Fig. 5:**
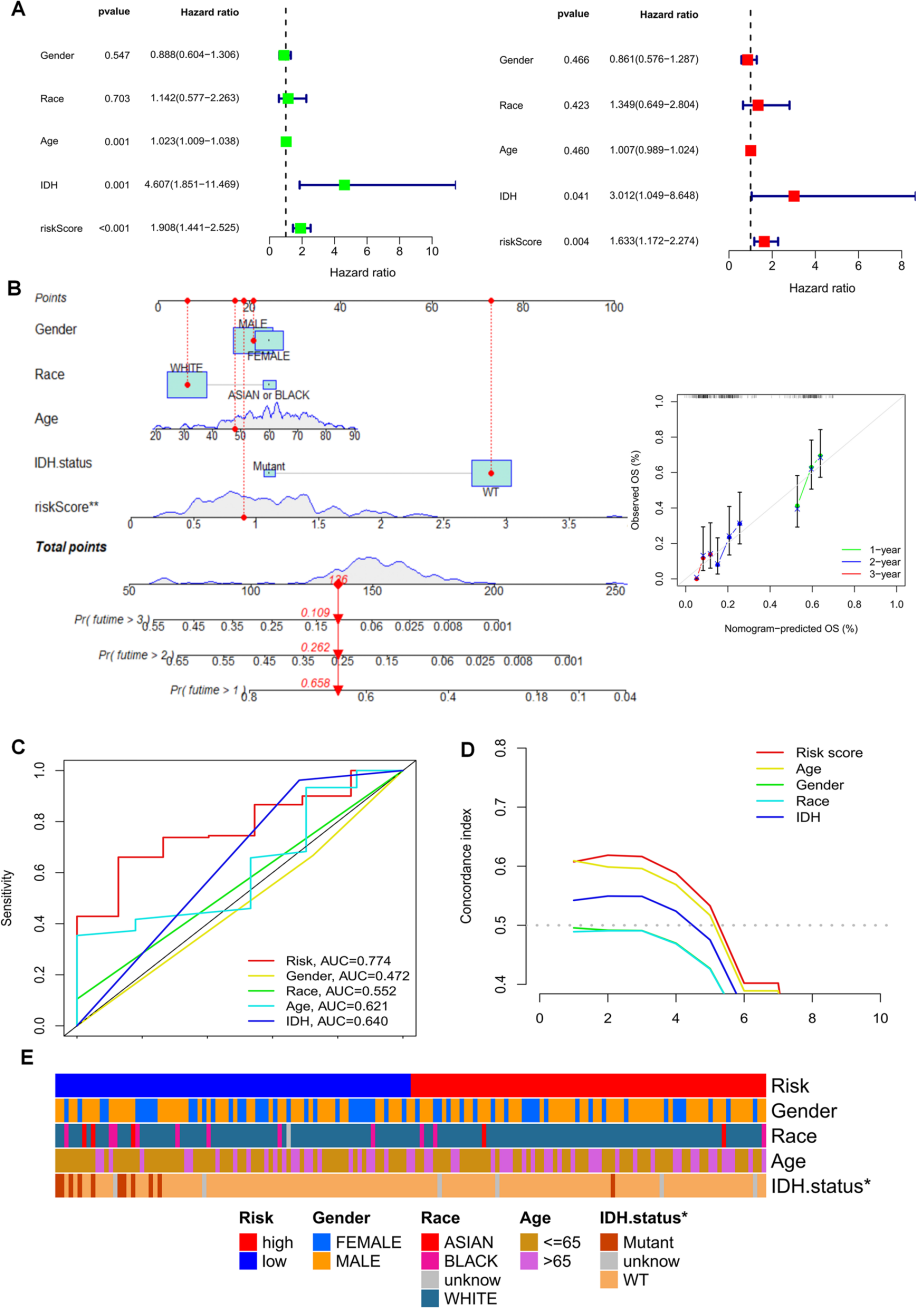
Prognostic value of endothelial cell expression-related signatures in the TCGA cohort. **A **Univariate and multivariate COX analysis for the riskscore and clinical features (including age, race, gender, and IDH state). **B **Nomogram for both the riskscore and clinical features to predict 1-, 2- and 3-year survival rates. The calibration curves test the consistency between the actual outcome and the predicted outcome at 1, 2, and 3 years. **C **AUC values for risk group and clinical features at three years. **D **The concordance index (C-index). **E **Bar chart of clinical characteristics under high and low-risk group

Univariate and multivariate Cox analyses revealed that risk scores could be an independent prognostic factor for GBM patients compared with other common clinical characteristics (Fig. 5A). Based on the risk regression model of the TCGA cohort, age, sex, race, and risk score were incorporated into the nomogram line graph to predict the survival status of patients with GBM as a whole. In the nomogram, risk scores for the endothelial cell hub gene had better predictive power than other clinicopathological features. The calibration curves also demonstrated acceptable agreement between actual and predicted survival at 1, 2, and 3 years (Fig. 5B), indicating that the risk model constructed based on the endothelial cell hub gene is reliable and can predict the prognosis of GBM patients well. The area under the curve (AUC) for the 1, 3, and 5-year risk class was higher than the AUC for the other clinicopathological features (Fig. 5C, Fig. S3), and the temporal c-index values for the risk class were similarly higher than for the other features (Fig. 5D). These results suggested that the prognostic functions of the four genetic features were quite reliable. The histogram of the chi-square test showed that the high-risk grouping was only associated with the mutational status of IDH (Fig. 5E).

**Fig. 6:**
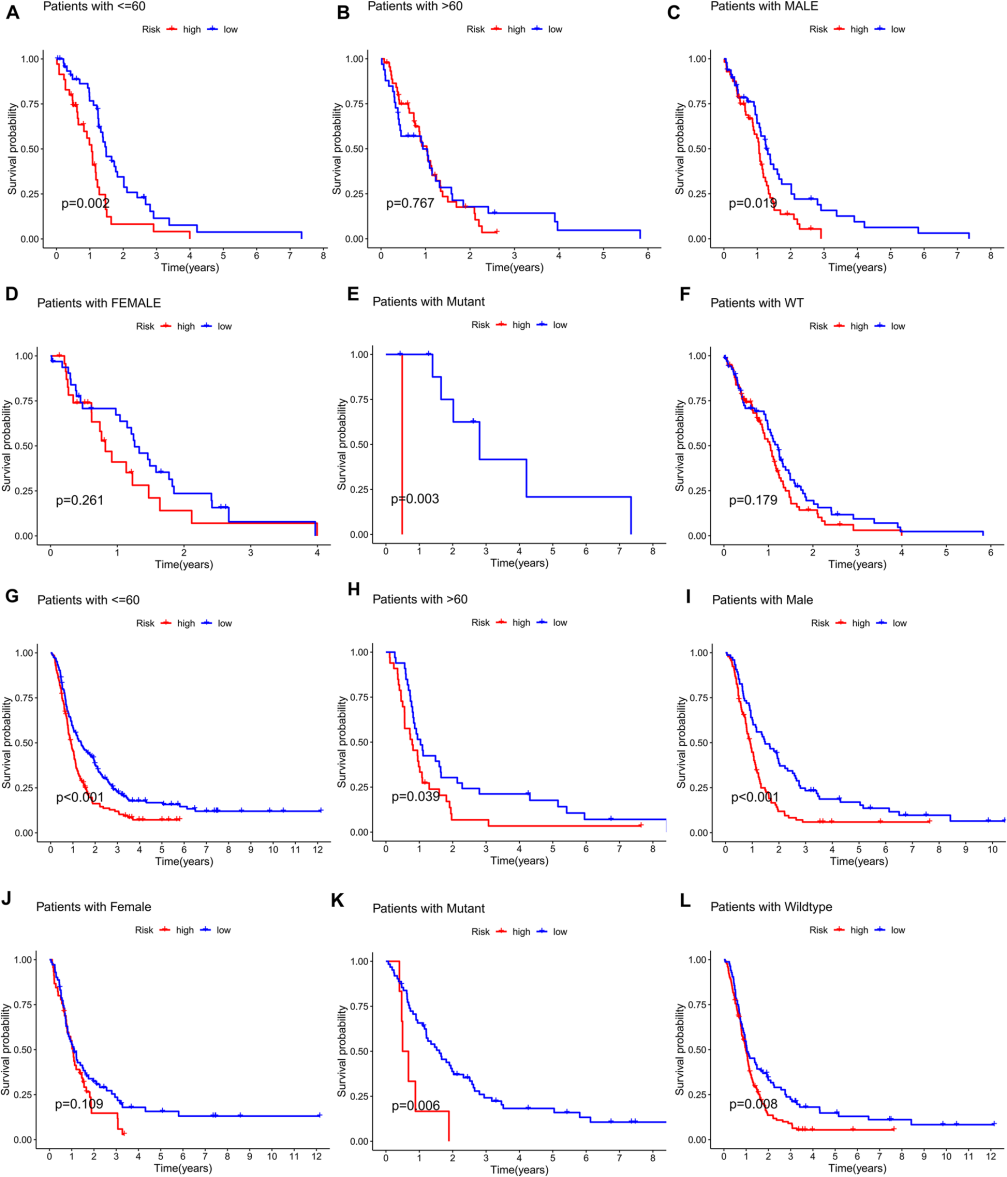
Kaplan-Meier survival curves for the low and high-risk groups in the TCGA and CGGA cohorts sorted by different clinicopathological variables.** A, B **Age, **C, D **sex, and **E, F **IDH mutations in the TCGA cohort. **G, H **Age, **I, J** sex, and **K, L **IDH mutations in the CGGA cohort

Also, GBM patients were grouped by age, gender, and IDH mutation status to investigate the relationship between risk characteristics and prognosis of GBM patients in these clinicopathological variables. For different staging, patients in the low-risk group of the TCGA and CGGA cohorts had significantly longer OS than those in the high-risk group (Fig. 6A-L). The differential results for the TCGA > 60 years group and the female subgroup may be due to poor prognosis in GBM and the limited number of patients. These results suggest that predictive characteristics may also predict the prognosis of GBM patients of different ages, genders, and IDH statuses.

### Mutation, immune function, enrichment analysis, and drug treatment analysis between the high-risk group and low-risk group

**Fig. 7:**
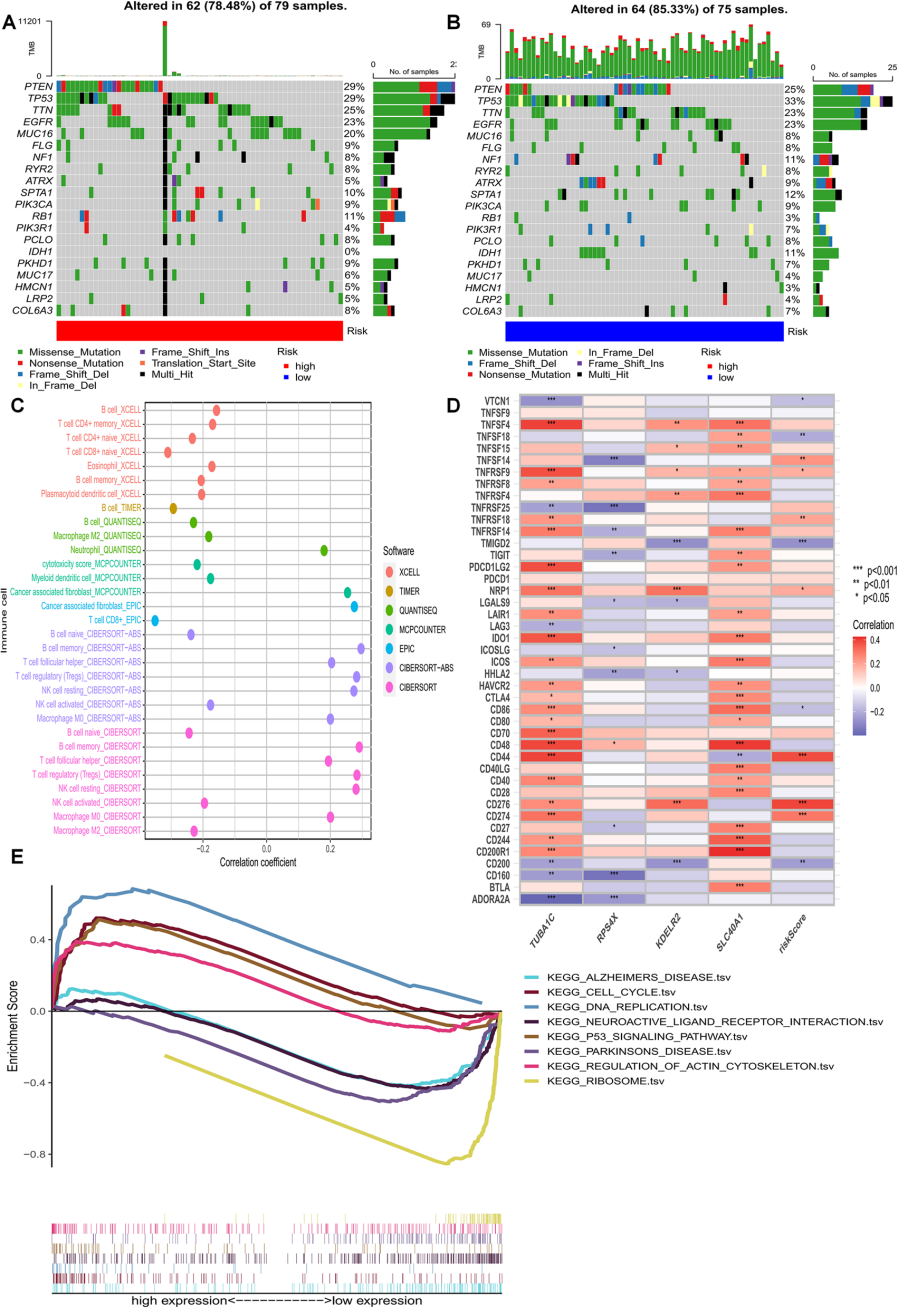
Mutation and immune correlation analysis based on risk score models. **A, B **Waterfall plots summarizing mutations in high- and low-risk populations. **C **The immune cell bubble of risk groups. **D **Heat map showing the correlation between immune checkpoint genes and TUBA1C, RPS4X, KDELR2, SLC40A1, and risk scores. **E **Gene set enrichment analysis of the top 8 pathways significantly enriched in the risk groups

Next, we generated two waterfall plots to explore the detailed gene mutations between the high-risk and low-risk groups, finding that TP53, TTN, and PTEN were the most commonly mutated genes in high-risk and low-risk groups (Fig. 7A, B). Next, we downloaded the immune cell infiltration data from the TCGA database from TIMER 2.0. Spearman correlation analysis revealed a correlation between risk scores and the abundance of immune cells in the GBM tumor microenvironment obtained by various algorithms. E.g., B cells in CIBERSORT, XCELL, and TIMER results were negatively correlated with risk scores (Fig. 7C). Next, using correlation heat maps, we investigated the correlation between the expression levels of the four genes and risk scores and genes associated with common ICIs in the model, respectively. The results showed that higher risk scores were significantly associated with the upregulation of CD276, CD274, and CD44 (Fig. 7D). In addition to this, we explored the correlation between risk score and tumor mutational load (TMB) and the difference in TMB between different risk groups (Fig. 4A), finding no significant association between risk score and TMB. Finally, using the R package “estimate”, we found no significant differences between stromal and immune scores in the high- and low-risk groups (Fig. 4B).

We applied TCIA to predict the susceptibility of patients with high and low-risk scores to immunotherapy. As shown in the figure, neither programmed cell death protein 1 (PD-1) nor cytotoxic t lymphocyte antigen 4 (CTLA4) was significant for treatment in the risk group (Fig. S4C), probably due to the very poor prognosis of GBM. We predicted the IC50 of all chemotherapeutic agents in the high- and low-risk score groups, finding that most of the agents such as AKT inhibitors and pabucirib exhibited a higher IC50 in patients with high-risk scores, thus suggesting that patients with high-risk scores may be more sensitive to these agents (p all < 0.05; Fig. 4D). In addition, we performed GSEA enrichment analysis between the TCGA high and low-risk datasets to assess the biological function of these genes. Using the gene set database MSigDB Collections (c2.cp.kegg.v7.4.symbols.gmt), we selected the eight most significant enriched signalling pathways based on normalized enrichment scores (NES) and *P* values (< 0.001) (Fig. 7E). p53 signaling pathway, cell cycle, DNA repair, and regulation of the actin cytoskeleton were enriched in the high-risk group, whereas the low-risk group had higher levels of Parkinson’s disease, ribosomal, Alzheimer’s disease, and neuroactive ligand-receptor interactions.

## Discussion

The process of angiogenesis implies the growth of new capillaries from pre-existing vessels. GBM is a highly vascularized tumour, and the growth of glioma is extremely dependent on the formation of new blood vessels [33]. Endothelial cells (ECs) dynamically modify their behavior during angiogenesis, eventually leading to differentiation, proliferation, migration, polarity, metabolism, and cell-cell communication changes. These modifications are assumed to integrate many external inputs; however, they also govern the ability of ECs to respond to environmental stimuli, such as up- or down-regulation of surface receptor expression [34]. In recent studies, TAM-derived factor (SEMA4D) has been shown to promote pericyte recruitment in neovascularization and cellular communication between glioma stem cell-derived perivascular cells and ECs, thus directly contributing to vascular stability in gliomas [35]. FAK proteins may increase angiogenesis in gliomas by triggering endothelial cell migration, according to research on ECs and angiogenesis. High-grade gliomas have higher FAK expression than low-grade gliomas and are associated with poorer survival [36]. As a result, anti-angiogenic therapies targeting ECs, which include inhibiting the proliferation of gliomas through angiogenesis-inhibiting factors and drugs to inhibit the formation of new tumor blood vessels, have gained increasing interest among researchers [37].

Characterization of ECs in normal brain tissue and GBM based on bulk RNAseq data is often limited [38]. In studies of ECs, it is often impossible to infer the effects of other cell types on account of GBM cell heterogeneity. In this study, we characterized the brain and GBM endothelial cells in more detail by integrating 10 × scRNA-seq and bulk RNA-seq data and used the mark gene of ECs to build a prognostic model for GBM patients, finding that the constructed prognostic model could effectively classify patients in the TCGA and CGGA cohorts into high- and low-risk groups. In addition, we explored survival status, clinical relevance, mutational status, and tumor immune infiltration in the different groups. Our results showed that higher risk scores were associated with a poorer prognosis, lower frequency of IDH mutations, and upregulation of immune checkpoints such as PD-L1 in patients. Therefore, patients with higher risk scores may be more likely to receive immunotherapy. In addition, we identified two different subtypes using the NMF algorithm. All patients in cluster 1 were immune C4 subtypes, which were associated with a worse prognosis [39]. We observed that the different subtypes had different prognostic and TME components. Group 1 was associated with poorer clinical outcomes and high infiltration levels of fibroblasts, whereas group 2 was associated with better clinical outcomes and high infiltration levels of cytotoxic lymphocytes. For example, fibroblasts can support tumor growth by consuming glucose [40].

We first identified mark genes in ECs by using single-cell sequencing, followed by LASSO and Cox regression analysis, which were used to identify four hub genes, i.e., TUBA1C, RPS4X, KDELR2, and SLC40A1, to model prognosis. TUBA1C is an isoform of alpha-microtubule protein that serves as a core component of the eukaryotic cytoskeleton and promotes cell division, formation, motility, and intracellular trafficking [41, 42]. In addition, the biological functions of microtubule proteins have been linked to cancer development, neurodevelopment, and neurodegenerative diseases [43]. In a recent study, TUBA1C expression was reported to be significantly higher in gliomas than in normal brain tissue, indicating a poorer prognosis. In addition, knockdown of TUBA1C also inhibited proliferation and migration of glioma cells, leading to apoptosis and G2/M phase arrest [44]. Studies on the oncogenic ribosomal protein S4 X-linked (RPS4X) have found that RPS4X increases cisplatin resistance after the depletion of specific small interfering RNAs. RPS4X is associated with ovarian cancer stage and its low expression is also associated with poor survival and disease progression [45]; however, there are still no reports on RPS4X in glioma. In hepatocellular carcinoma, RPS4X is required for SLFN11 inactivation in the mTOR signaling pathway [46]. Interestingly, the KDEL receptor (KDELR2) can also target and promote the growth of HIF1a through the mTOR signaling pathway to guide glioblastoma [47]. KDELR2 knockdown reduces cell viability, promotes G1 phase cell cycle arrest, and induces apoptosis. Furthermore, KDELR2 can regulate cellular function in glioma cells by targeting CCND1 [48]. Solute carrier family 40 member 1 (SLC40A1) is a gene encoding an iron transporter protein. Previous studies in multiple myeloma and ovarian cancer have shown that SLC40A1 inhibits tumor cell growth and reduces resistance to chemotherapy [49, 50]. Only one recent bioinformatics study has suggested that the ferroptosis suppressor SLC40A1 is associated with immunosuppression in gliomas and that acetaminophen may exert antitumor effects in GBM by modulating SLC40A1-induced death [51].

Our results showed that the developed prognostic model exhibited independent predictive power in predicting OS in GBM patients. We found no significant difference between the high-risk and low-risk groups in terms of gene mutations such as TP53 and PTEN; however, the high-risk group did not have any IDH mutations. According to the 2016 WHO classification, there is a significant difference between IDH mutant GBM and IDH wild-type GBM, which has a poorer prognosis [52]. This further validates the reliability of our model. In addition, we investigated the relationship between risk scores, TMB values, and PD-L1 expression levels. Disappointingly, higher risk scores were not significantly correlated with higher TMB values (Fig. S3A). A key mediator of immunosuppression in GBM is PD-L1, and although only a fraction of GBM cells express PD-L1, PD-L1 expression in the tumor microenvironment is deficient [53].

Finally, immune checkpoint blockade treatment may be more effective in individuals with higher risk ratings. The developed prognostic model might be used as a predictive biomarker for immunotherapy patients. The CGGA external validation cohort was also employed to confirm the model’s accuracy in predicting OS in these individuals. Nonetheless, the present study has some limitations. To begin with, all of the presented findings are based on bioinformatic studies and require further experimental confirmation. To corroborate our findings, we created an endothelial cell-based biomarker that will need to be tested in large-scale clinical studies.

## Conclusion

The present study constructed and validated a prognostic model for GBM by integrating 10× scRNA-seq and bulk RNA-seq data. Higher risk scores were significantly associated with poorer survival outcomes, with almost zero IDH mutation rates and upregulation of immune checkpoints such as PD-L1 and CD276. Our prognostic model may be used as a potential biomarker for risk stratification and treatment response prediction in GBM patients.

## Supplementary Information

**Additional file 1: Table S1**. The detailed clinical characteristics of patients in the TCGA and CGGA cohorts.

**Additional file 2: Figure S1.** (A)After cell quality control (QC), 102412 cells were identified. (B) Top 2000 highly variable genes.

**Additional file 3: Figure S2**. (A)The role of the first 100 genes in cell development. (B-D)Infer the cellular communication network by calculating the possibility of communication.

**Additional file 4: Figure S3.** AUC values for prognostic characteristics and clinical features at 1(A) and 5(B) years.

**Additional file 5: Figure S4**. (A)Correlation of risk scores and tumour mutational load (TMB). (B) Association of risk score with stromal scoring and immune scoring based on the results of the ESTIMATE algorithm. (C) Relative probability of risk score to ctla -4 antibody and pd -1/ PD-L1 antibody response. (D) Calculation of IC50 values based on AKT inhibitors,synonyms,pabuciclib, mTOR inhibitors and Mitomycin C for patients in high- and low-score risk groups to evaluate the sensitivity of chemotherapeutic agents.

## Data Availability

The datasets analyzed for this study were obtained from the UCSC Xena website (https://xenabrowser.net/datapages/) and CGGA dataset(http://www.cgga.org.cn/index.jsp).

## References

[1] Siegel RL, Miller KD, Jemal A (2019). Cancer statistics, 2019. CA Cancer J Clin.

[2] Ostrom QT (2014). The epidemiology of glioma in adults: a “state of the science” review. Neuro Oncol.

[3] Bangalore YC (2020). A novel fully automated MRI-based deep-learning method for classification of IDH mutation status in brain gliomas. Neuro Oncol.

[4] Chiocca EA, et al. Regulatable interleukin-12 gene therapy in patients with recurrent high-grade glioma: results of a phase 1 trial. Sci Transl Med. 2019;11(505):eaaw5680. 10.1126/scitranslmed.aaw5680. 10.1126/scitranslmed.aaw5680 PMC7286430 31413142

[5] Miroshnikova YA (2016). Tissue mechanics promote IDH1-dependent HIF1alpha-tenascin C feedback to regulate glioblastoma aggression. Nat Cell Biol.

[6] Sarkaria JN (2018). Is the blood-brain barrier really disrupted in all glioblastomas? A critical assessment of existing clinical data. Neuro Oncol.

[7] Qu S, Li S, Hu Z (2020). Upregulation of Piezo1 Is a Novel Prognostic Indicator in Glioma Patients. Cancer Manag Res.

[8] Alkins R (2016). Early treatment of HER2-amplified brain tumors with targeted NK-92 cells and focused ultrasound improves survival. Neuro Oncol.

[9] Xiao D (2021). A ferroptosis-related prognostic risk score model to predict clinical significance and immunogenic characteristics in glioblastoma multiforme. Oxid Med Cell Longev.

[10] Wang G (2022). Angiogenesis-Related Gene Signature-Derived Risk Score for Glioblastoma: Prospects for Predicting Prognosis and Immune Heterogeneity in Glioblastoma. Front Cell Dev Biol.

[11] Chen Z (2020). Identification of differentially expressed genes in lung adenocarcinoma cells using single-cell RNA sequencing not detected using traditional RNA sequencing and microarray. Lab Invest.

[12] Wang J, Gareri C, Rockman HA (2018). G-Protein-Coupled Receptors in Heart Disease. Circ Res.

[13] Yang F (2021). Uncovering a Distinct Gene Signature in Endothelial Cells Associated With Contrast Enhancement in Glioblastoma. Front Oncol.

[14] Schaaf MB (2019). Autophagy in endothelial cells and tumor angiogenesis. Cell Death Differ.

[15] Langenkamp E (2015). Elevated expression of the C-type lectin CD93 in the glioblastoma vasculature regulates cytoskeletal rearrangements that enhance vessel function and reduce host survival. Cancer Res.

[16] Ma J, Waxman DJ (2008). Combination of antiangiogenesis with chemotherapy for more effective cancer treatment. Mol Cancer Ther.

[17] Zhang L (2018). IDH mutation status is associated with distinct vascular gene expression signatures in lower-grade gliomas. Neuro Oncol.

[18] Carmeliet P (2003). Angiogenesis in health and disease. Nat Med.

[19] Macosko EZ (2015). Highly Parallel Genome-wide Expression Profiling of Individual Cells Using Nanoliter Droplets. Cell.

[20] Habicht J (2019). UNC-45A is preferentially expressed in epithelial cells and binds to and co-localizes with interphase MTs. Cancer Biol Ther.

[21] Becht E, et al., Dimensionality reduction for visualizing single-cell data using UMAP. Nat Biotechnol. 2018. 10.1038/nbt.4314. 10.1038/nbt.4314 30531897

[22] Aran D (2019). Reference-based analysis of lung single-cell sequencing reveals a transitional profibrotic macrophage. Nat Immunol.

[23] Griss J (2020). ReactomeGSA - Efficient Multi-Omics Comparative Pathway Analysis. Mol Cell Proteomics.

[24] Borcherding N (2019). Single-Cell Profiling of Cutaneous T-Cell Lymphoma Reveals Underlying Heterogeneity Associated with Disease Progression. Clin Cancer Res.

[25] Jin S (2021). Inference and analysis of cell-cell communication using CellChat. Nat Commun.

[26] Wang C, et al., Identification of prognostic candidate genes in breast cancer by integrated bioinformatic analysis. J Clin Med, 2019;8(8). 10.3390/jcm8081160 PMC6723760 31382519

[27] Tamborero D (2018). A Pan-cancer Landscape of Interactions between Solid Tumors and Infiltrating Immune Cell Populations. Clin Cancer Res.

[28] Zhang Z (2018). Time-varying covariates and coefficients in Cox regression models. Ann Transl Med.

[29] Sun S (2020). Development and validation of an immune-related prognostic signature in lung adenocarcinoma. Cancer Med.

[30] Charoentong P (2017). Pan-cancer Immunogenomic Analyses Reveal Genotype-Immunophenotype Relationships and Predictors of Response to Checkpoint Blockade. Cell Rep.

[31] Ghosh MK (2019). The interrelationship between cerebral ischemic stroke and glioma: a comprehensive study of recent reports. Signal Transduct Target Ther.

[32] Bentley RT (2017). Dogs are man’s best friend: in sickness and in health. Neuro Oncol.

[33] Levin VA, Ellingson BM (2018). Understanding brain penetrance of anticancer drugs. Neuro Oncol.

[34] Jeong HW (2017). Transcriptional regulation of endothelial cell behavior during sprouting angiogenesis. Nat Commun.

[35] Zhu C (2017). The contribution of tumor-associated macrophages in glioma neo-angiogenesis and implications for anti-angiogenic strategies. Neuro Oncol.

[36] Brown NF (2018). A study of the focal adhesion kinase inhibitor GSK2256098 in patients with recurrent glioblastoma with evaluation of tumor penetration of [11 C]GSK2256098. Neuro Oncol.

[37] Xu R (2016). Molecular and Clinical Effects of Notch Inhibition in Glioma Patients: A Phase 0/I Trial. Clin Cancer Res.

[38] Dieterich LC (2012). Transcriptional profiling of human glioblastoma vessels indicates a key role of VEGF-A and TGFbeta2 in vascular abnormalization. J Pathol.

[39] Ye X (2021). ALOX5AP Predicts Poor Prognosis by Enhancing M2 Macrophages Polarization and Immunosuppression in Serous Ovarian Cancer Microenvironment. Front Oncol.

[40] Schworer S, Vardhana SA, Thompson CB (2019). Cancer Metabolism Drives a Stromal Regenerative Response. Cell Metab.

[41] Nieuwenhuis J, Brummelkamp TR (2019). The Tubulin Detyrosination Cycle: Function and Enzymes. Trends Cell Biol.

[42] Janke C, Magiera MM (2020). The tubulin code and its role in controlling microtubule properties and functions. Nat Rev Mol Cell Biol.

[43] Roll-Mecak A (2020). The Tubulin Code in Microtubule Dynamics and Information Encoding. Dev Cell.

[44] Gui S (2021). TUBA1C expression promotes proliferation by regulating the cell cycle and indicates poor prognosis in glioma. Biochem Biophys Res Commun.

[45] Tsofack SP (2013). Low expression of the X-linked ribosomal protein S4 in human serous epithelial ovarian cancer is associated with a poor prognosis. BMC Cancer.

[46] Zhou C (2020). SLFN11 inhibits hepatocellular carcinoma tumorigenesis and metastasis by targeting RPS4X via mTOR pathway. Theranostics.

[47] Liao Z (2019). KDELR2 Promotes Glioblastoma Tumorigenesis Targeted by HIF1a via mTOR Signaling Pathway. Cell Mol Neurobiol.

[48] Mao H (2020). KDELR2 is an unfavorable prognostic biomarker and regulates CCND1 to promote tumor progression in glioma. Pathol Res Pract.

[49] Kong Y (2019). Ferroportin downregulation promotes cell proliferation by modulating the Nrf2-miR-17-5p axis in multiple myeloma. Cell Death Dis.

[50] Wu J (2020). miR-194-5p inhibits SLC40A1 expression to induce cisplatin resistance in ovarian cancer. Pathol Res Pract.

[51] Deng S (2021). Ferroptosis Suppressive Genes Correlate with Immunosuppression in Glioblastoma. World Neurosurg.

[52] Liu YQ (2019). Gene Expression Profiling Stratifies IDH-Wildtype Glioblastoma With Distinct Prognoses. Front Oncol.

[53] Lamano JB (2019). Glioblastoma-Derived IL6 Induces Immunosuppressive Peripheral Myeloid Cell PD-L1 and Promotes Tumor Growth. Clin Cancer Res.

