# Supplementary material for: Expression of hub genes of endothelial cells in glioblastoma-A prognostic model for GBM patients integrating single-cell RNA sequencing and bulk RNA sequencing
Source: BMC Cancer. 2022 Dec 6;22:1274. doi: 10.1186/s12885-022-10305-z (PMC9724299; doi:10.1186/s12885-022-10305-z)
Supplement: Supplementary file 1 — Additional file 1: Table S1. The detailed clinical characteristics of patients in the TCGA and CGGA cohorts. [file 12885_2022_10305_MOESM1_ESM.doc]

**Table S1. The detailed clinical characteristics of patients in the TCGA and CGGA**

**cohorts.**

| Variables | TCGA cohort(n=169) | CGGA cohort(n=388) |
| --- | --- | --- |
| Gender |  |  |
| Female | 59 | 153 |
| Male | 109 | 235 |
| NA | 1 | 0 |
| Age |  |  |
| <=60 | 87 | 319 |
| >60 | 81 | 69 |
| NA | 1 | 0 |
| IDH state |  |  |
| Mutant | 12 | 90 |
| WT | 149 | 288 |
| NA | 8 | 10 |
| 1p/19q |  |  |
| codel | 0 | 20 |
| non-codel | 161 | 334 |
| NA | 8 | 34 |
