# Supplementary figures and images for: Expression of hub genes of endothelial cells in glioblastoma-A prognostic model for GBM patients integrating single-cell RNA sequencing and bulk RNA sequencing
Source: BMC Cancer. 2022 Dec 6;22:1274. doi: 10.1186/s12885-022-10305-z (PMC9724299; doi:10.1186/s12885-022-10305-z)

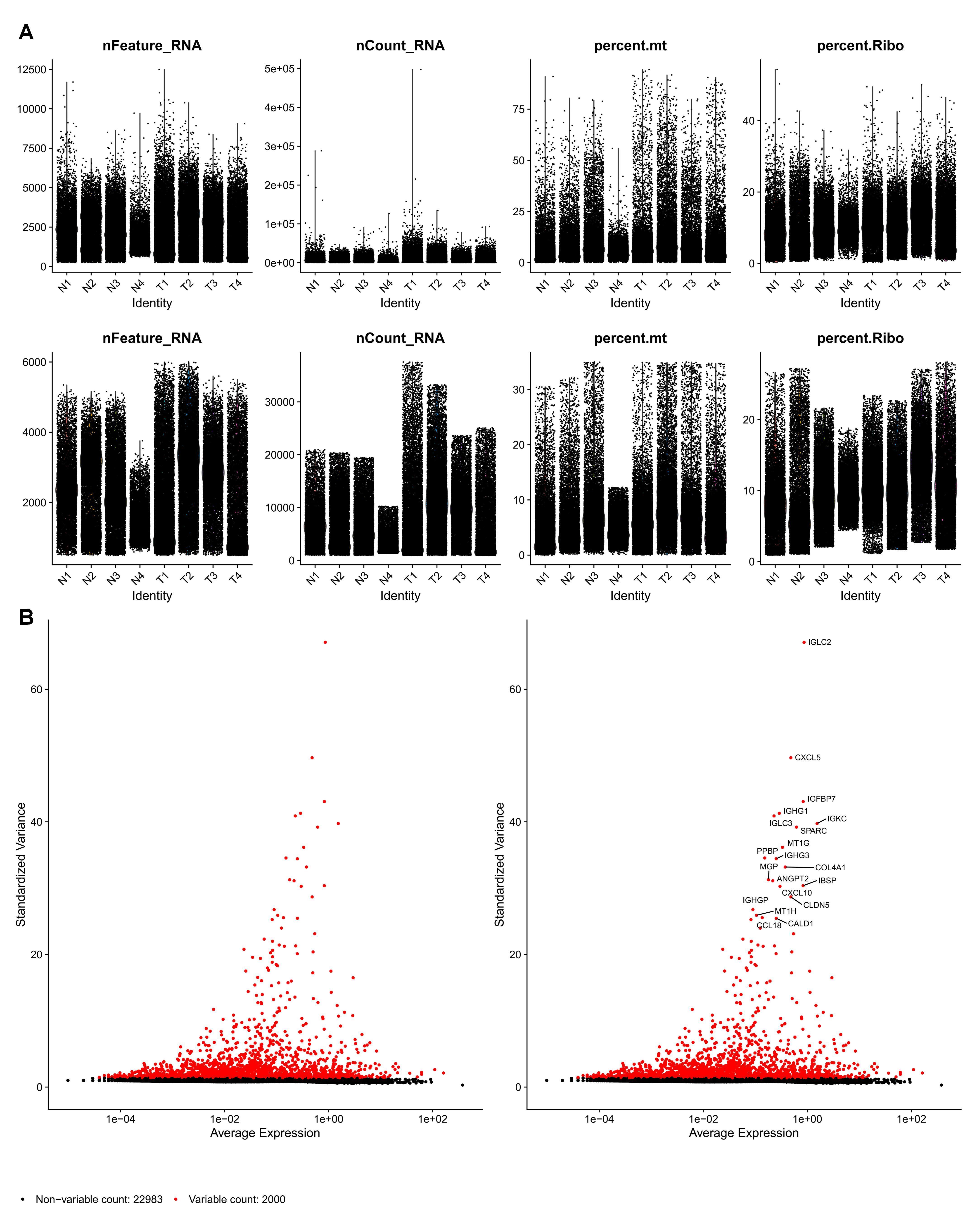

Supplement: Supplementary file 2 — Additional file 2: Figure S1. (A)After cell quality control (QC), 102412 cells were identified. (B) Top 2000 highly variable genes. [file 12885_2022_10305_MOESM2_ESM.jpg]

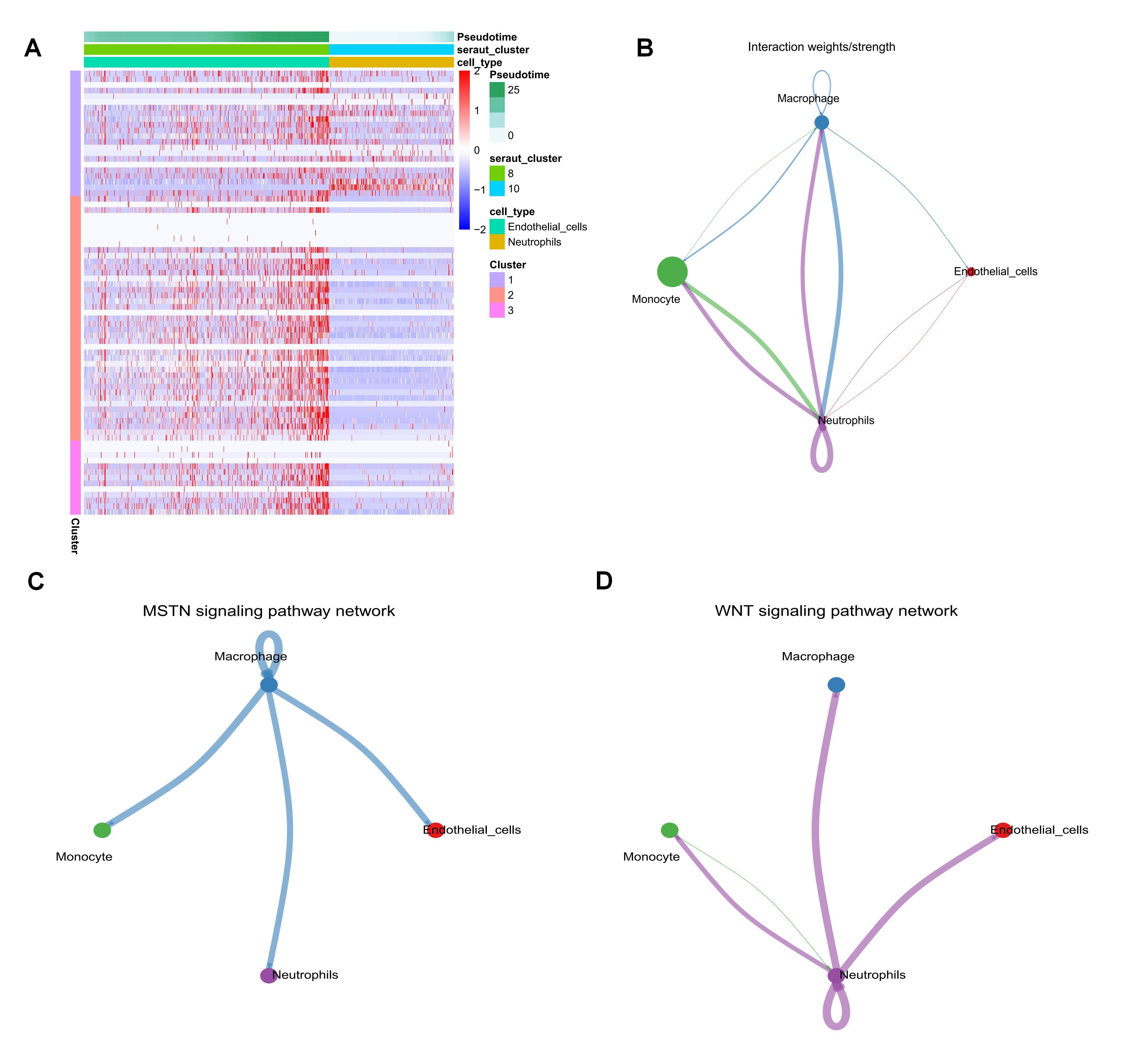

Supplement: Supplementary file 3 — Additional file 3: Figure S2. (A)The role of the first 100 genes in cell development. (B-D)Infer the cellular communication network by calculating the possibility of communication. [file 12885_2022_10305_MOESM3_ESM.jpg]

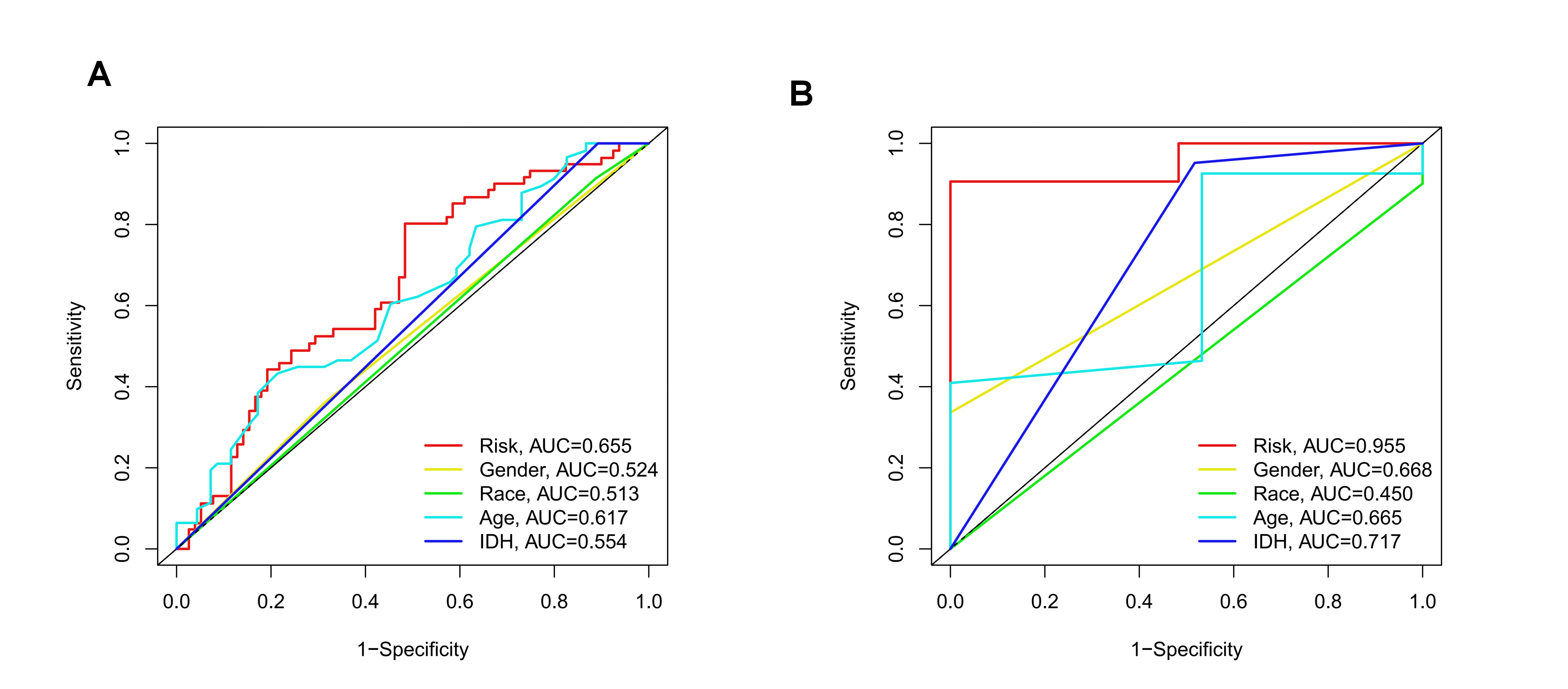

Supplement: Supplementary file 4 — Additional file 4: Figure S3. AUC values for prognostic characteristics and clinical features at 1(A) and 5(B) years. [file 12885_2022_10305_MOESM4_ESM.png]

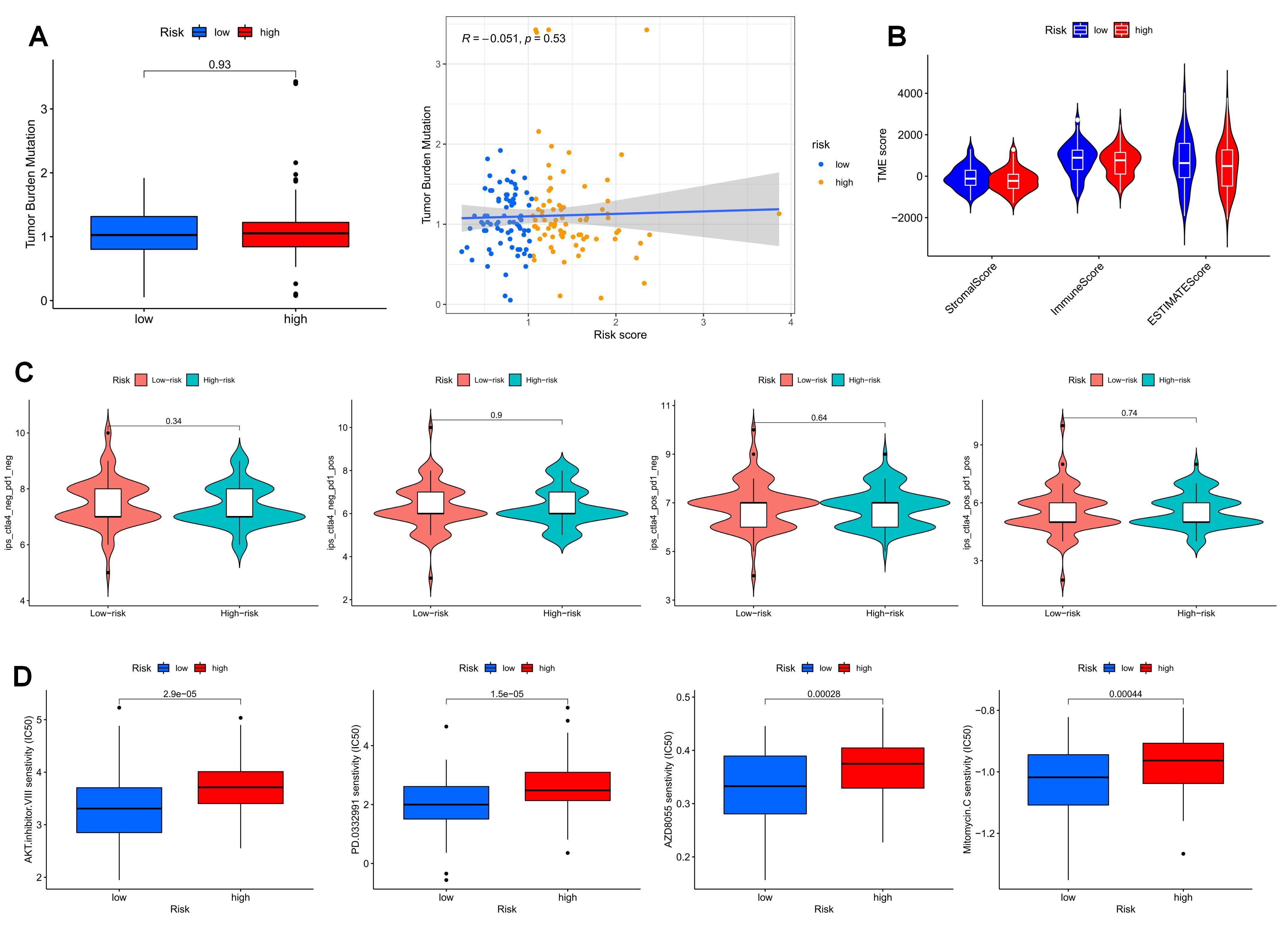

Supplement: Supplementary file 5 — Additional file 5: Figure S4. (A)Correlation of risk scores and tumour mutational load (TMB). (B) Association of risk score with stromal scoring and immune scoring based on the results of the ESTIMATE algorithm. (C) Relative probability of risk score to ctla -4 antibody and pd -1/ PD-L1 antibody response. (D) Calculation of IC50 values based on AKT inhibitors,synonyms,pabuciclib, mTOR inhibitors and Mitomycin C for patients in high- and low-score risk groups to evaluate the sensitivity of chemotherapeutic agents. [file 12885_2022_10305_MOESM5_ESM.jpg]
